# KIF4A-KIF4B Paralog as a Prognostic Biomarker in Lung Adenocarcinoma

**DOI:** 10.3390/medicina62071424

**Published:** 2026-07-22

**Authors:** Hyun-Soo Park, Jun-Chae Lee, Hyowon Hong, Jae-Ho Lee

**Affiliations:** 1 Medical Course, School of Medicine, Keimyung University, Daegu 42601, Republic of Korea; clover73731@naver.com (H.-S.P.); chai1703@naver.com (J.-C.L.); 2 Department of Anatomy, School of Medicine, Keimyung University, Daegu 42601, Republic of Korea

**Keywords:** lung adenocarcinoma, KIF4A, KIF4B, TCGA

## Abstract

*Background and Objectives*: Lung adenocarcinoma (LUAD) is a clinically heterogeneous malignancy, and reliable prognostic biomarkers are still needed. Kinesin family members 4A and 4B (KIF4A and KIF4B) are mitosis-related motor proteins with potential functional overlap as paralogs. However, their coordinated prognostic significance in LUAD has not been systematically investigated. *Materials and Methods*: Transcriptomic and clinical data from The Cancer Genome Atlas (TCGA) were analyzed to evaluate the expression patterns, clinicopathologic associations, and prognostic significance of KIF4A and KIF4B in LUAD. Correlation analysis, Kaplan–Meier survival analysis, and Cox proportional hazards regression analyses were performed. In addition, a combined paralog score and four-group expression model were evaluated. An independent LUAD cohort from the Gene Expression Omnibus (GEO; GSE81089) was analyzed for external validation. *Results*: KIF4A and KIF4B expression showed a strong positive correlation (r = 0.767, *p* < 0.001), and both genes were positively correlated with EGFR, KRAS, BRAF, MKI67, and PCNA expression. High KIF4A expression was significantly associated with age, sex, smoking status, pathologic stage, N stage, and T stage, whereas high KIF4B expression was significantly associated with age and pathologic stage. Kaplan–Meier analysis demonstrated that high expression of both KIF4A and KIF4B was associated with poorer overall survival. In Cox regression analyses, elevated expression of both genes remained significantly associated with unfavorable overall survival in univariable and multivariable models. However, when both genes were simultaneously included in the same Cox model, their individual prognostic effects were attenuated, suggesting substantial overlap in prognostic information. By contrast, the combined paralog score remained independently associated with poor overall survival. In four-group analysis, only patients with concurrent high expression of both KIF4A and KIF4B showed significantly worse overall survival compared with the low/low group. External validation demonstrated generally consistent survival patterns, although the prognostic associations were attenuated after multivariable adjustment. *Conclusions*: KIF4A and KIF4B expression showed substantial prognostic overlap in LUAD, and their concurrent high expression was associated with poorer overall survival. These findings suggest that KIF4A/KIF4B co-expression may serve as a candidate prognostic indicator that warrants validation in independent clinical cohorts and functional studies.

## 1. Introduction

Lung cancer remains one of the most commonly diagnosed malignancies and the leading cause of cancer-related mortality worldwide [1]. According to recent global cancer statistics, approximately 2.2 million new lung cancer cases and 1.8 million deaths were reported in 2020, accounting for 11.4% of all newly diagnosed cancers and 18.0% of cancer-related deaths globally [2]. These high incidence and mortality rates of lung cancer highlight the need for identifying biomarkers for its early diagnosis, prognostic assessment, and the selection of appropriate therapeutic strategies.

Histologically, lung cancer is broadly classified into non-small cell lung cancer (NSCLC) and small cell lung cancer (SCLC). NSCLC accounts for approximately 85% of all lung cancer cases and mainly consists of lung adenocarcinoma (LUAD) and lung squamous cell carcinoma (LUSC), which account for approximately 40% and 25% of lung cancers, respectively [3,4]. As the most common histological subtype, LUAD shows its distinct molecular and clinical characteristics compared with other NSCLC subtypes, which supports the need to consider these subtypes as biologically and clinically distinct entities [5,6].

LUAD is characterized by multiple oncogenic driver mutations that activate key signaling pathways regulating tumor proliferation and survival. The most frequently altered genes include EGFR, KRAS, and BRAF, which converge on the MAPK signaling pathway [5,7]. These driver events are typically mutually exclusive and define distinct molecular subgroups of LUAD with different clinical outcomes and therapeutic responses [5]. In addition, mutations of the tumor suppressor gene TP53, known as a key regulator of cell-cycle checkpoints, are among the most common alterations in LUAD contributing to aberrant cell-cycle progression and chromosomal instability [5,8]. These findings suggest that not only oncogenic signaling but also dysregulation of cell-cycle control and mitotic progression may contribute to LUAD progression.

Mitotic processes are highly related to the interactions between microtubules and motor proteins to ensure accurate chromosome segregation during cell division [9]. Kinesins are microtubule-dependent molecular motors that play essential roles such as intracellular transport, spindle organization, and chromosome movement during mitosis [9,10].

Among these proteins, kinesin family member 4A (KIF4A) and kinesin family member 4B (KIF4B) belong to the kinesin-4 subfamily and share substantial structural similarity [11]. KIF4A has been reported to participate in mitotic chromosome condensation and segregation, and spindle organization [12]. Also, KIF4B has been implicated in the regulation of spindle dynamics and cytokinesis during mitosis [13]. Given their structural homology and reported mitosis-related functions, KIF4A and KIF4B may show coordinated expression in proliferative tumor states. However, despite their potential functional linkage, the coordinated clinical and prognostic significance of KIF4A and KIF4B in lung adenocarcinoma has not yet been systematically investigated. Therefore, evaluating their expression patterns may help determine whether their co-expression is associated with clinical outcomes beyond the information provided by either gene alone.

Therefore, the present study aimed to investigate the prognostic significance of KIF4A and KIF4B expression in LUAD. Using transcriptomic and clinical data from The Cancer Genome Atlas (TCGA), we analyzed the expression patterns of KIF4A and KIF4B, assessed their association with patient survival, and evaluated whether their combined expression pattern was associated with overall survival in LUAD.

## 2. Materials and Methods

### 2.1. TCGA Data Analysis

We utilized TCGA-based data for this study. We initially screened the prognostic significance of KIF4A and KIF4B across TCGA cancer types using OncoLnc, and LUAD was selected because both genes demonstrated significant prognostic associations (Supplementary Figures S1 and S2). Among these, LUAD showed significant associations for both genes and was therefore selected for further detailed analysis. To investigate the clinical significance of KIF4A and KIF4B, mRNA expression data were extracted together with those of major LUAD driver genes, including EGFR, KRAS, TP53, and BRAF, for correlation analysis. A total of 492 patients with LUAD were included in the initial dataset. In Cox regression analyses, cases with incomplete data for variables included in each model were excluded. Therefore, the final sample size was 482 patients in each Cox model. This study followed the publication guidelines for the use of TCGA data sets (https://portal.gdc.cancer.gov/) (accessed on 14 October 2025).

### 2.2. GEO Data Analysis

External validation was performed using an independent NSCLC cohort (GSE81089) retrieved from the Gene Expression Omnibus (GEO) database. After identifying LUAD cases based on the provided histology annotation, patients were dichotomized into high- and low-expression groups according to the median expression value of each gene for Kaplan–Meier survival analysis. Because the GEO cohort was generated using a different expression platform from the TCGA RNA-sequencing dataset, log2-transformed gene expression values were standardized (z-score transformation) before Cox regression analysis.

### 2.3. Statistical Analysis

Statistical analyses were performed using SPSS software (version 25.0; IBM Corp., Armonk, NY, USA) and R software (version 4.5.2; R Foundation for Statistical Computing, Vienna, Austria). Associations between categorical expression groups and clinicopathological characteristics were analyzed using the chi-square test. Tumor staging was determined according to the seventh edition of the American Joint Committee on Cancer (AJCC) staging system. Spearman’s correlation analysis was performed using SPSS software to evaluate the association between the continuous expression levels of KIF4A and KIF4B. Additional correlation analyses were conducted to assess the relationships of KIF4A and KIF4B with major LUAD driver genes and canonical proliferation-associated marker genes. For Cox proportional hazards regression analyses, mRNA expression values were log2-transformed using the formula log2 (expression + 1) to reduce skewness in the expression distribution. A paralog score was then calculated as the sum of the log2-transformed expression levels of KIF4A and KIF4B. Univariate and multivariate Cox proportional hazards regression analyses were performed using R software to evaluate the prognostic significance of KIF4A expression, KIF4B expression, and the paralog score for overall survival (OS). In univariate Cox models, KIF4A expression, KIF4B expression, and the paralog score were analyzed individually as continuous variables. Multivariate Cox models were adjusted for clinicopathological variables including age at initial pathologic diagnosis, gender, pathologic stage and smoking status. Hazard ratios (HRs) and 95% confidence intervals (CIs) were calculated. A *p*-value < 0.05 was considered statistically significant. In addition, combined KIF4A/KIF4B expression status was assessed using a four-group categorical classification (high/high, high/low, low/high, and low/low).

## 3. Results

### 3.1. Correlation Analysis Between KIF4A, KIF4B, LUAD Driving Genes, and Canonical Proliferation-Associated Genes

Correlation analysis revealed that KIF4A expression was positively correlated with MKI67 (r = 0.793, *p* < 0.001), PCNA (r = 0.536, *p* < 0.001), EGFR (r = 0.183, *p* < 0.001), KRAS (r = 0.115, *p* = 0.010), and BRAF (r = 0.249, *p* < 0.001), whereas it showed a modest negative correlation with TP53 (r = -0.111, *p* = 0.014) in LUAD (Table 1). Similarly, KIF4B expression was positively correlated with MKI67 (r = 0.657, *p* < 0.001), PCNA (r = 0.381, *p* < 0.001), EGFR (r = 0.133, *p* = 0.003), KRAS (r = 0.107, *p* = 0.017), and BRAF (r = 0.192, *p* < 0.001), whereas no significant correlation was observed between KIF4B and TP53 (r = −0.020, *p* = 0.650). Notably, KIF4A and KIF4B expression levels were strongly positively correlated with each other, (r = 0.767, *p* < 0.001), supporting their coordinated expression in LUAD.

### 3.2. Clinical Characteristics of KIF4A and KIF4B Expressions

In LUAD, KIF4A and KIF4B expressions were significantly associated with several clinicopathologic parameters (Table 2). High KIF4A expression was significantly associated with age (*p* = 0.030), gender (*p* < 0.001), smoking status (*p* = 0.043), pathologic stage (*p* = 0.002), N stage (*p* = 0.007), and T stage (*p* = 0.026). Similarly, high KIF4B expression was significantly associated with age (*p* = 0.047) and pathologic stage (*p* = 0.027). Borderline associations were observed for gender (*p* = 0.070) and N stage (*p* = 0.090).

### 3.3. Survival Analysis of KIF4A and KIF4B Expressions

**Figure 1:**
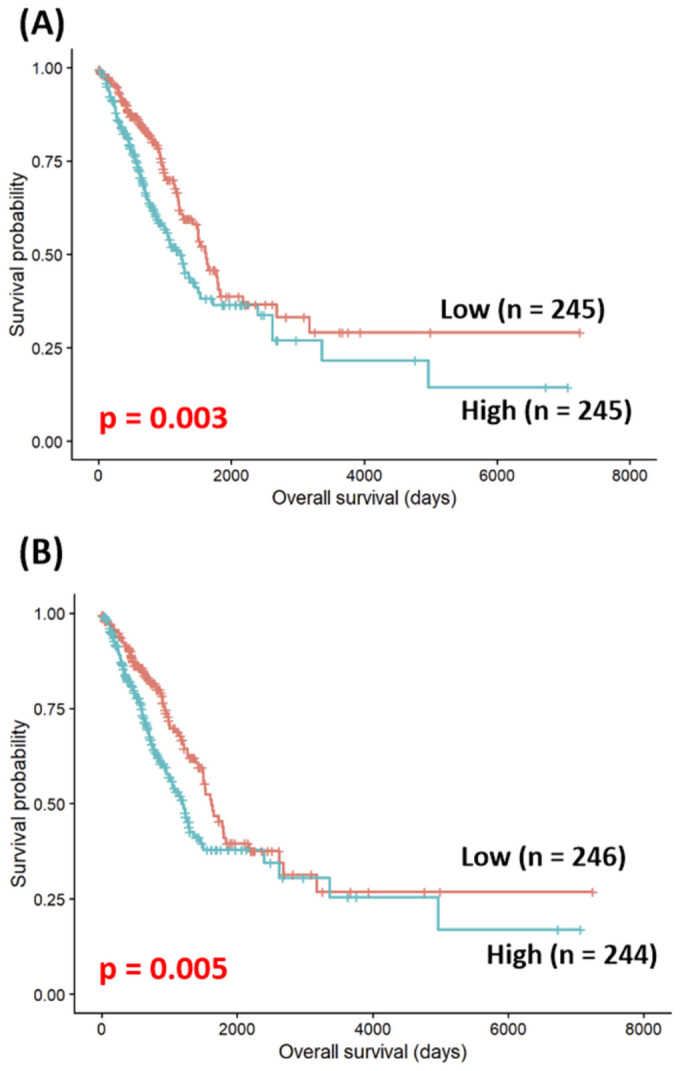
TCGA data of Overall survival analysis in lung adenocarcinoma according to KIF4A expression (**A**) and KIF4B expression (**B**).

Kaplan–Meier survival analysis demonstrated that patients with high KIF4A expression had significantly poorer overall survival than those with low expression (2300.580 ± 320.502 vs. 3024.818 ± 350.492 days; χ^2^ = 8.889, *p* = 0.003; Figure 1A). Likewise, patients with high KIF4B expression had significantly poorer overall survival than those with low expression (2459.074 ± 340.114 vs. 2933.095 ± 345.317 days; χ^2^ = 7.749, *p* = 0.005; Figure 1B).

### 3.4. Cox Regression Analysis of KIF4A, KIF4B, and Combined Biomarkers in LUAD

To ensure consistency of the study population, cases with missing values in any of the variables included in the Cox regression models were excluded. Univariable and multivariable Cox regression analyses were performed in the same complete-case cohort (*n* = 482, events = 176). Expression of both KIF4A and KIF4B was significantly associated with poorer overall survival in LUAD in univariable Cox regression analysis. These associations remained significant after adjustment for age at initial pathologic diagnosis, gender, pathologic stage, and smoking status in multivariable Cox regression analysis, indicating that increased expression of both genes may serve as independent prognostic biomarkers in LUAD.

Univariable Cox regression analysis showed that both KIF4A and KIF4B expression were significantly associated with poorer overall survival in LUAD (KIF4A: HR = 1.254, 95% CI = 1.126–1.397, *p* < 0.001; KIF4B: HR = 1.323, 95% CI = 1.157–1.514, *p* < 0.001; Table 3). In multivariable Cox regression analysis adjusted for age at initial pathologic diagnosis, gender, pathologic stage, and smoking status, both genes remained significantly associated with poorer overall survival (KIF4A: HR = 1.218, 95% CI = 1.092–1.359, *p* < 0.001; KIF4B: HR = 1.262, 95% CI = 1.099–1.449, *p* < 0.001).

To further examine whether KIF4A and KIF4B exerted independent prognostic effects beyond their shared expression pattern, both genes were simultaneously included in the same multivariable Cox regression model. In this model, the association of KIF4A with overall survival was attenuated to borderline significance (HR = 1.160, 95% CI = 0.985–1.366, *p* = 0.075), whereas KIF4B was no longer significantly associated with overall survival (HR = 1.088, 95% CI = 0.881–1.344, *p* = 0.431). These findings suggest substantial overlap in the prognostic information provided by KIF4A and KIF4B, consistent with their strong positive correlation (Table 4).

To evaluate the combined prognostic significance of KIF4A and KIF4B, a paralog score was calculated by summing the log2-transformed expression levels of the two genes (Table 5). In univariable Cox regression analysis, the paralog score was significantly associated with poorer overall survival in LUAD (HR = 1.151, 95% CI = 1.081–1.226, *p* < 0.001). This association remained significant in multivariable Cox regression analysis adjusted for age at initial pathologic diagnosis, gender, pathologic stage, and smoking status (HR = 1.129, 95% CI = 1.058–1.204, *p* < 0.001).

Combined expression groups were defined according to the median expression levels of KIF4A and KIF4B in the complete-case cohort used for Cox regression analysis. Consistent with the continuous correlation analysis, categorical grouping based on median expression also demonstrated a significant association between KIF4A and KIF4B expression status (*p* < 0.001), with most patients classified into the high/high and low/low groups (Table 6). For the group labels, the first term refers to KIF4A expression status and the second term refers to KIF4B expression status. The reference group was Low/Low. Multivariable Cox regression analysis was adjusted for age at initial pathologic diagnosis, gender, pathologic stage, and smoking status.

In multivariable Cox regression analysis, the high/low and low/high groups were not significantly associated with overall survival compared with the low/low group (Table 7). However, the high/high group was associated with poorer overall survival than the low/low group (adjusted HR = 1.606, 95% CI = 1.136–2.269, *p* = 0.007), suggesting that simultaneous overexpression of KIF4A and KIF4B has stronger prognostic significance than isolated high expression of either gene.

### 3.5. External Validation of the KIF4A–KIF4B Prognostic Model in an Independent GEO Cohort

**Figure 2:**
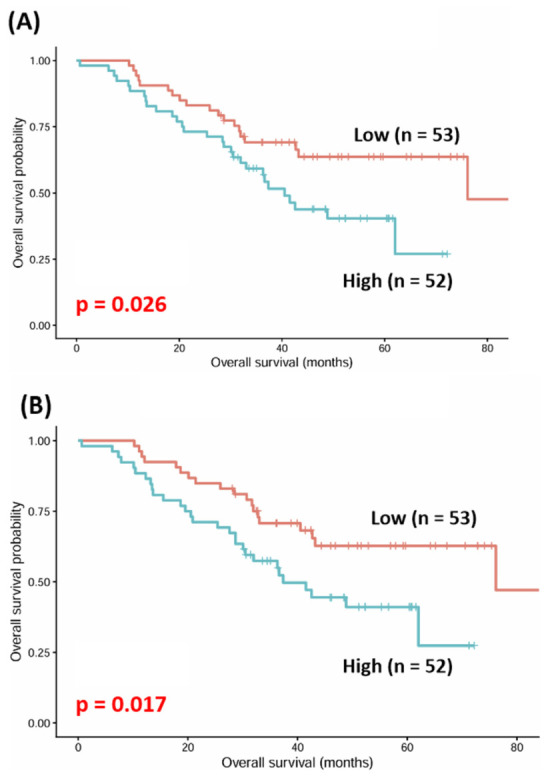
GEO data of overall survival in an independent LUAD cohort according to KIF4A expression (**A**) and KIF4B expression (**B**).

To validate the prognostic significance of the KIF4A–KIF4B paralog axis, an independent LUAD cohort from the GEO database (GSE81089) was analyzed. Kaplan–Meier survival analysis in the independent GSE81089 cohort similarly demonstrated that high KIF4A expression was significantly associated with poorer overall survival than low expression (1426.089 ± 147.358 vs. 1896.734 ± 132.305 days; χ^2^ = 4.975, *p* = 0.026; Figure 2A). Likewise, high KIF4B expression was associated with worse overall survival than low expression (1413.806 ± 149.644 vs. 1905.002 ± 130.197 days; χ^2^ = 5.742, *p* = 0.017; Figure 2B).

In univariable Cox regression analysis, both KIF4A and KIF4B expression were significantly associated with poorer overall survival (KIF4A: HR = 1.440, 95% CI = 1.086–1.910, *p* = 0.011; KIF4B: HR = 1.376, 95% CI = 1.059–1.788, *p* = 0.017; Table 8). After adjustment for clinical covariates, neither KIF4A nor KIF4B remained statistically significant (KIF4A: HR = 1.276, 95% CI = 0.940–1.732, *p* = 0.119; KIF4B: HR = 1.230, 95% CI = 0.925–1.638, *p* = 0.155; Table 8). Likewise, when both genes were simultaneously included in the same multivariable Cox regression model, neither KIF4A (HR = 1.356, 95% CI = 0.573–3.207, *p* = 0.488) nor KIF4B (HR = 0.928, 95% CI = 0.407–2.115, *p* = 0.858) showed an independent association with overall survival (Table 9).

The prognostic performance of the combined KIF4A–KIF4B paralog score was further evaluated in the external cohort. In univariable Cox regression analysis, the paralog score was significantly associated with poorer overall survival (HR = 1.412, 95% CI = 1.078–1.847, *p* = 0.012; Table 10). In multivariable Cox regression analysis adjusted for clinical covariates, the prognostic effect was attenuated and did not remain statistically significant (HR = 1.241, 95% CI = 0.926–1.662, *p* = 0.148; Table 10).

## 4. Discussion

Our findings indicate that KIF4A and KIF4B expression levels are strongly correlated and show overlapping associations with overall survival in LUAD. Rather than demonstrating two independent prognostic effects, the results suggest that the two genes may reflect a shared transcriptional pattern associated with proliferative tumor biology. This interpretation is supported by three complementary observations. First, the two genes showed a strong positive correlation. Second, their expression was associated with adverse clinicopathologic features. Third, their prognostic effect was most evident when they were assessed together rather than separately. In particular, the simultaneous Cox model attenuated the individual effects of KIF4A and KIF4B, whereas the paralog score remained significant and only the high/high subgroup showed worse survival compared with the low/low group. Collectively, these results suggest that the clinical relevance of KIF4A and KIF4B lies less in independent prognostic contribution and more in their concurrent high expression as a co-expression pattern associated with unfavorable clinical outcomes.

KIF4A and KIF4B showed strong co-expression, whereas their correlations with EGFR, KRAS, TP53, and BRAF expression levels were weak to modest. These exploratory associations should therefore not be interpreted as evidence of direct pathway regulation, mutation-specific effects, or functional interaction with canonical LUAD driver genes. Instead, they may reflect broader variation in tumor transcriptional states. Consistent with this interpretation, both KIF4A and KIF4B showed stronger correlations with the canonical proliferation-associated marker genes MKI67 and PCNA. In particular, KIF4A and KIF4B showed strong positive correlations with MKI67, and moderate positive correlations with PCNA were also observed. These findings support the interpretation that KIF4A/KIF4B co-expression is more closely related to a cell-cycle or proliferation-associated transcriptional program than to direct association with specific LUAD driver gene expression.

Biologically, this interpretation is plausible given the established role of the kinesin-4 family in mitotic regulation. KIF4A is involved in chromosome condensation and chromosome organization, and it also regulates spindle microtubule dynamics and cytokinetic progression [12,14,15]. These functions place KIF4A close to the core machinery required for accurate cell division. In this context, high KIF4A expression in LUAD may indicate more than simple proliferation. Previous studies in LUAD have shown that KIF4A is functionally linked to increased proliferation and migration, supporting the view that elevated KIF4A expression may reflect biologically meaningful tumor progression [16,17]. Although KIF4B has been less extensively characterized than KIF4A, prior studies describe it as an intronless and highly similar paralog of KIF4A that shares key structural features [18,19]. KIF4B depletion has also been reported to cause mitotic defects similar to those observed after KIF4A loss [13]. These prior observations provide biological context for the present co-expression findings; however, they do not establish a functional role for KIF4B in LUAD and should be interpreted cautiously.

The clinicopathologic associations further support this interpretation. High KIF4A expression was significantly associated with age, sex, pathologic stage, N stage, and T stage, whereas high KIF4B expression was significantly associated with age and pathologic stage, with borderline associations for sex and N stage. Importantly, the strongest and most consistent signals involved overall pathologic stage and local or nodal tumor burden rather than distant metastasis. The stronger clinicopathologic associations observed for KIF4A than for KIF4B may indicate differences in the magnitude of their expression-outcome associations. Therefore, these clinicopathologic findings should be interpreted as supportive associations rather than evidence that KIF4A is functionally dominant or that KIF4B has a complementary mechanistic role in LUAD.

The Cox analyses are particularly important because they clarify the prognostic meaning of the paralog concept. Individually, both KIF4A and KIF4B were associated with poorer overall survival in univariable and multivariable models. However, when both genes were entered simultaneously, their individual significance was lost or attenuated, indicating that the prognostic information carried by the two genes is substantially overlapping rather than independent. This interpretation is reinforced by the paralog score, which remained significantly associated with survival after adjustment for clinical covariates. Furthermore, in the four-group analysis, only the high/high group showed a statistically significant association with poorer survival compared with the low/low group. These findings suggest that concurrent high expression of KIF4A and KIF4B may identify a subgroup of LUAD patients with less favorable clinical outcomes.

To further evaluate the robustness of our findings, we performed external validation using an independent LUAD cohort from the GEO database (GSE81089). Kaplan–Meier survival analyses consistently demonstrated poorer overall survival in patients with high KIF4A or KIF4B expression, reproducing the overall survival patterns observed in the TCGA cohort. Similarly, univariable Cox regression analyses showed significant associations between KIF4A, KIF4B, and the KIF4A–KIF4B paralog score and overall survival. Although these associations were attenuated after multivariable adjustment, this may be attributable to the relatively small sample size of the external cohort and differences in available clinical variables. Nevertheless, the external cohort provided supportive evidence for the overall prognostic pattern of the KIF4A–KIF4B paralog observed in the TCGA cohort.

Several limitations should be acknowledged. First, although the prognostic associations were evaluated in an external cohort, the present study was primarily based on retrospective transcriptomic analyses and may still be affected by residual confounding. In particular, the Cox regression models were not adjusted for the mutation status of major LUAD driver genes, including EGFR, KRAS, TP53, and BRAF. Therefore, the observed associations between KIF4A/KIF4B expression and clinical outcomes should not be interpreted as independent of all molecular characteristics of LUAD. Second, the analyses were based on bulk mRNA expression data and did not assess protein expression, cellular localization, cell-type-specific expression patterns, or pathway activity within the tumor microenvironment. Third, although both KIF4A and KIF4B have reported mitosis-related functions in prior experimental studies, the present analysis does not directly establish whether their co-expression contributes to chromosome organization, spindle function, cytokinesis, or tumor progression in LUAD. Therefore, the observed co-expression pattern should be regarded as a candidate prognostic pattern, and future mutation-adjusted analyses, protein-level validation, and functional experiments are required to determine its biological and clinical relevance.

## 5. Conclusions

In this TCGA-based retrospective analysis, high expression of KIF4A and KIF4B, particularly concurrent high expression, was associated with poorer overall survival in LUAD. The attenuation of individual associations in simultaneous Cox models suggests that the two genes provide substantially overlapping prognostic information. These findings support KIF4A/KIF4B co-expression as a candidate prognostic pattern rather than establishing a functional paralog axis or direct molecular mechanism. External validation, mutation-adjusted analyses, protein-level assessment, and functional experiments are needed before potential clinical utility can be determined.

## Figures and Tables

**Table 1:** Correlation analysis of KIF4A, KIF4B, major LUAD driver genes, and canonical proliferation-associated genes.

		KIF4A	KIF4B	EGFR	KRAS	TP53	BRAF	MKI67	PCNA
KIF4A	r	1	0.767	0.183	0.115	−0.111	0.249	0.793	0.536
	*p*		<0.001	<0.001	0.010	0.014	<0.001	<0.001	<0.001
KIF4B	r	0.767	1	0.133	0.107	−0.020	0.192	0.657	0.381
	*p*	<0.001		0.003	0.017	0.650	<0.001	<0.001	<0.001
EGFR	r	0.183	0.133	1	0.011	−0.123	0.061	0.213	−0.029
	*p*	<0.001	0.003		0.810	0.007	0.177	<0.001	0.518
KRAS	r	0.115	0.107	0.011	1	−0.057	0.080	0.153	0.053
	*p*	0.010	0.017	0.810		0.209	0.078	<0.001	0.239
TP53	r	−0.111	−0.020	−0.123	−0.057	1	−0.003	−0.070	0.084
	*p*	0.014	0.650	0.007	0.209		0.940	0.122	0.064
BRAF	r	0.249	0.192	0.061	0.080	−0.003	1	0.230	0.091
	*p*	<0.001	<0.001	0.177	0.078	0.940		<0.001	0.043
MKI67	r	0.793	0.657	0.213	0.153	−0.070	0.230	1	0.413
	*p*	<0.001	<0.001	<0.001	<0.001	0.122	<0.001		<0.001
PCNA	r	0.536	0.381	−0.029	0.053	0.084	0.091	0.413	1
	*p*	<0.001	<0.001	0.518	0.239	0.064	0.043	<0.001	

**Table 2:** Clinical characteristics of KIF4A and KIF4B genes in LUAD.

	KIF4A			KIF4B		
	High	Low	*p*-Value	High	Low	*p*-Value
**Age**						
≤65	130	106	0.030	129	107	0.047
>65	115	139		116	138	
**Gender**						
Female	114	152	<0.001	123	143	0.070
Male	131	93		122	102	
**Smoking Status**						
No	69	90	0.043	74	85	0.289
Yes	177	156		172	161	
**Pathologic stage**						
Stage I	111	151	0.002	115	147	0.027
Stage II	68	49		68	49	
Stage III	48	31		45	34	
Stage IV	16	8		14	10	
**M stage**						
M0	159	163	0.102	161	161	0.431
M1	16	8		14	10	
**N stage**						
N0	142	174	0.007	148	168	0.090
N1	55	38		53	40	
N2	42	26		39	29	
N3	2	0		2	0	
**T stage**						
T1	66	98	0.026	71	93	0.100
T2	143	117		135	125	
T3	24	21		28	17	
T4	10	8		10	8	

**Table 3:** Univariate and multivariate Cox regression analyses of KIF4A and KIF4B for overall survival in LUAD.

Variable	Univariable HR (95% CI)	*p*-Value	Multivariable HR (95% CI)	*p*-Value
KIF4A	1.254 (1.126–1.397)	<0.001	1.218 (1.092–1.359)	<0.001
KIF4B	1.323 (1.157–1.514)	<0.001	1.262 (1.099–1.449)	< 0.001

**Table 4:** Cox regression analyses of simultaneous KIF4A/KIF4B expression models for overall survival in LUAD.

Gene in Simultaneous Model	Multivariable HR (95% CI)	*p*-Value
KIF4A	1.160 (0.985–1.366)	0.075
KIF4B	1.088 (0.881–1.344)	0.431

**Table 5:** Cox regression analyses of the paralog score for overall survival in LUAD.

Variable	Univariable HR (95% CI)	*p*-Value	Multivariable HR (95% CI)	*p*-Value
Paralog Score	1.151 (1.081–1.226)	<0.001	1.129 (1.058–1.204)	<0.001

**Table 6:** Distribution of four combined KIF4A/KIF4B expression groups in the complete-case LUAD cohort used for Cox regression analysis.

	High KIF4A Expression	Low KIF4A Expression	*p*-Value
High KIF4B expression	189	52	<0.001
Low KIF4B expression	52	189	

**Table 7:** Multivariable Cox regression analysis of four combined KIF4A/KIF4B expression groups for overall survival in LUAD.

Comparison with Low/Low	Multivariable HR (95% CI)	*p*-Value
High / Low	1.045 (0.608–1.798)	0.873
Low/High	0.978 (0.549–1.742)	0.940
High/High	1.606 (1.136–2.269)	0.007

**Table 8:** Cox regression analyses of KIF4A and KIF4B expression for overall survival in the external LUAD cohort (GSE81089).

Variable	Univariable HR (95% CI)	*p*-Value	Multivariable HR (95% CI)	*p*-Value
KIF4A	1.440 (1.086–1.910)	0.011	1.276 (0.940–1.732)	0.119
KIF4B	1.376 (1.059–1.788)	0.017	1.230 (0.925–1.638)	0.155

**Table 9:** Multivariable Cox regression analysis of the simultaneous KIF4A/KIF4B expression model in the external LUAD cohort (GSE81089).

Gene in Simultaneous Model	Multivariable HR (95% CI)	*p*-Value
KIF4A	1.356 (0.573–3.207)	0.488
KIF4B	0.928 (0.407–2.115)	0.858

**Table 10:** Cox regression analyses of the KIF4A–KIF4B paralog score in the external LUAD cohort (GSE81089).

Variable	Univariable HR (95% CI)	*p*-Value	Multivariable HR (95% CI)	*p*-Value
Paralog Score	1.412 (1.078–1.847)	0.012	1.241 (0.926–1.662)	0.148

## Data Availability

The data generated in the present study may be requested from the corresponding author.

## Supplementary Materials

The following supporting information can be downloaded at: https://www.mdpi.com/article/10.3390/medicina62071424/s1, Figure S1. Prognostic ranking of KIF4A expression across various cancer types; Figure S2. Prognostic ranking of KIF4B expression across various cancer types.
