# Supplementary material for: KIF4A-KIF4B Paralog as a Prognostic Biomarker in Lung Adenocarcinoma
Source: Medicina (Kaunas). 2026 Jul 22;62(7):1424. doi: 10.3390/medicina62071424 (PMC13413802; doi:10.3390/medicina62071424)
Supplement: Supplementary file 1 [file medicina-62-01424-s001.zip › medicina-4358202-supplementary.docx]

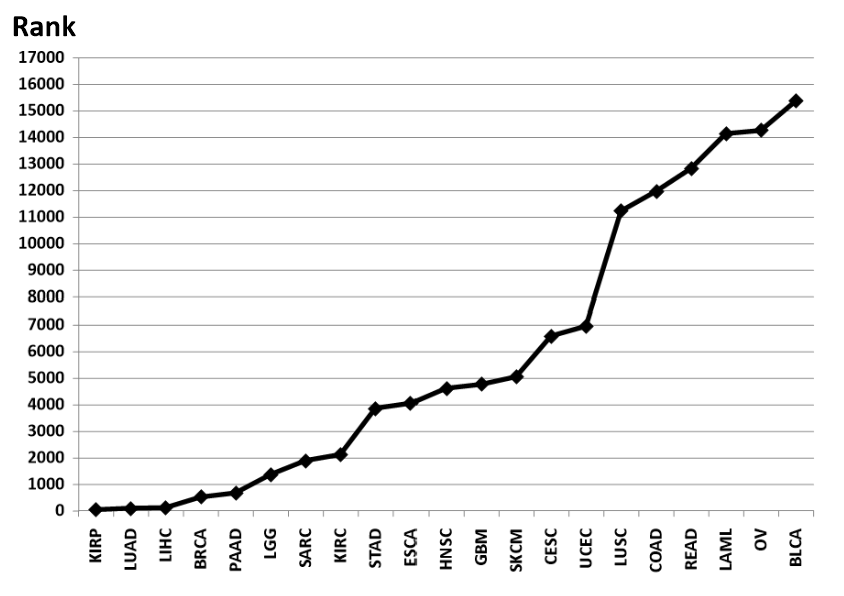


Supplementary Figure S1. Prognostic ranking of KIF4A expression across various cancer types


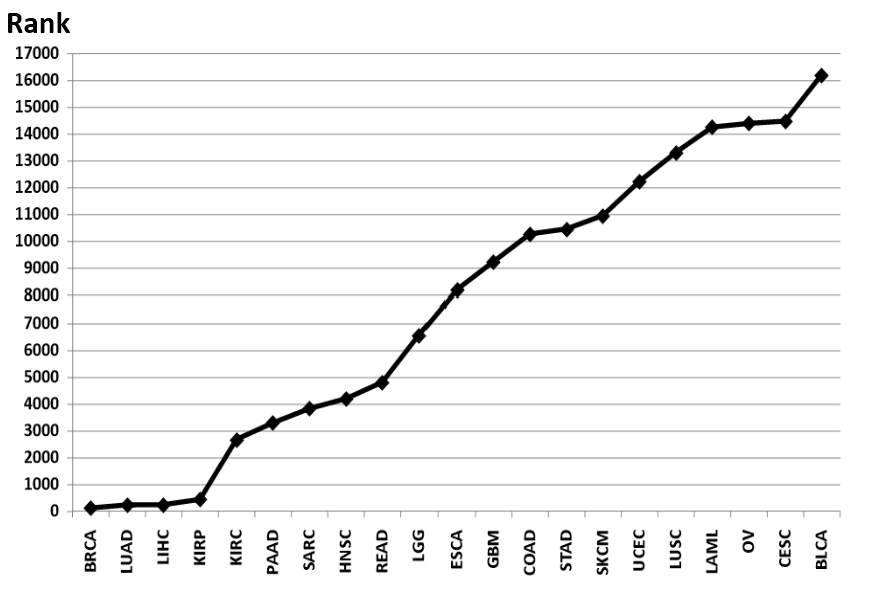


Supplementary Figure S2. Prognostic ranking of KIF4B expression across various cancer types
